# Personalized Nursing and Health Care: Advancing Positive Patient Outcomes in Complex and Multilevel Care Environments

**DOI:** 10.3390/jpm12111801

**Published:** 2022-11-01

**Authors:** Riitta Suhonen, Minna Stolt, David Edvardsson

**Affiliations:** 1 Department of Nursing Science, University of Turku, 20014 Turku, Finland; 2 Welfare Services Division, Turku University Hospital, 20014 Turku, Finland; 3 School of Nursing & Midwifery, La Trobe University, Melbourne, VIC 3086, Australia

This Special Issue of the *Journal of Personalised Medicine* invited manuscripts that further establish the current state of science relating to personalized nursing and health care. We welcomed manuscripts that highlight and further the knowledge base conceptually, instrumentally, observationally and experimentally, with sound theoretical and methodological underpinnings and implications for research, theory and clinical work in the disciplines of nursing, medicine, allied health and beyond. As there has been a rapid development in the academic literature over the last ten years in terms of papers relating to individualization, personalization, patient-, client-, consumer- and person-centredness, with work on conceptual, instrumental, observational and experimental levels, this theme seemed timely and relevant [1,2].

The individuality of care and services is essential for the realisation of healthcare quality, ethical obligations and the development of a deeper understanding of user perspectives necessary for health care, health policy development and increasing patient choice [3]. Healthcare systems in countries should be based on the comprehensive need assessment of individual clients [4,5] and patients to provide individualised, personalised or tailored care [6]. Personalized medical care should not only improve the patient’s situation by providing the right diagnosis, prevention or treatment; it also needs to be tailored according to individual characteristics, situation, context, and environment to support people’s health power, health careers and thus, their self-management and independent living [7,8]. This is highly important as health care systems have taken responsibility for care that also warrants the increasing responsibility of people’s own self-management [9,10]. In order to support self-care, an individualised assessment of care needs is needed; furthermore, individual client’s and patient’s active participation in determining care and co-designing services is necessary [11]. Self-care is of vital importance for sustainable healthcare; however, it is not sufficiently emphasised. Knowing the clients and patients, assessing their individual needs and responding to these needs in an individualised manner has been found effective, and even cost-effective. Such initiative requires individualised or tailored interventions that are effective in care delivery. However, the complex multilevel interplay between care and service networks for patients, especially for older people, is rarely studied.

Individualised treatment and nursing care is an activity carried out by professionals and provides the perception of personalised care; it can act as an indicator of person-centeredness, requiring person-centred behaviour and other forms of competence [12,13]. Such care does not appear in and of itself, and health professionals need to support new-comers to in providing individualised or person-centred care for citizens [14]. Individualised care and patient-centeredness can be seen as a process or specific set of nursing or other care activities which produce positive patient outcomes [15]. However, there is a need to change the shift from system and professional-centred activities to patient-centeredness and orientation to build usable and effective care options for different groups of people, including individualised environments that support self-management and independence [16,17]. However, health system reforms have not necessarily taken into account the processes from the client’s point of view and there is also a lack of studies focusing on the care environment of older people. There is also a need to increase the multidisciplinarity of care assessment, planning, delivery and evaluation, where different professions and disciplines work with the person at the centre of care and treatment; there has never been a better time to do this than now due to developments in precision medicine, personalized treatment and care, digitalisation [18] and consumer/community participation. All these contemporary trends and support systems can facilitate increased agency on behalf of patients/persons in need of care and can facilitate the health literacy and agency needed to individualise care directed by the person with health needs [19]. However, some further developments are needed to improve the ‘individual/person literacy’ on behalf of health care professionals and how this information is collected, shared and implemented in care decisions [20]. Standards and structures can also restrict the sharing of agency, power, expertise and accountability of healthcare decisions and actions, particularly in the specialized healthcare space. However, as this Special issue demonstrates, there are rapid developments in the space of personalised medicine and care, which indicate a promising future ahead.

We hope to provide an interesting and comprehensive reading experience with this Special issue. We thank all the authors and editorial office professionals for their contributions to this Special Issue; we also thank the journal and publication platform for providing us with the opportunity to collect multi-disciplinary work covering various important approaches. This Special Issue highlights the scientific advancements in the field as well as meaningful results that can be used to advance healthcare systems.

## Data Availability

Not applicable.
